# Age-Stratified Model to Assess Health Outcomes of COVID-19 Vaccination Strategies, Ghana

**DOI:** 10.3201/eid2902.221098

**Published:** 2023-02

**Authors:** Sylvia K. Ofori, Jessica S. Schwind, Kelly L. Sullivan, Gerardo Chowell, Benjamin J. Cowling, Isaac Chun-Hai Fung

**Affiliations:** Harvard T.H. Chan School of Public Health, Boston, Massachusetts, USA (S.K. Ofori);; Georgia Southern University Jiann-Ping Hsu College of Public Health, Statesboro, Georgia, USA (S.K. Ofori, K.L. Sullivan, I.C.-H. Fung);; Georgia Southern University Institute for Health Logistics and Analytics, Statesboro (J.S. Schwind);; Georgia State University School of Public Health, Atlanta, Georgia, USA (G. Chowell);; WHO Collaborating Centre for Infectious Disease Epidemiology and Control, The University of Hong Kong School of Public Health, Pokfulam, Hong Kong (B.J. Cowling)

**Keywords:** COVID-19, Age-stratified model, vaccination, Ghana, coronavirus disease, SARS-CoV-2, severe acute respiratory syndrome coronavirus 2, viruses, respiratory infections, zoonoses, vaccine-preventable diseases

## Abstract

We assessed the effect of various COVID-19 vaccination strategies on health outcomes in Ghana by using an age-stratified compartmental model. We stratified the population into 3 age groups: <25 years, 25–64 years, and ≥65 years. We explored 5 vaccination optimization scenarios using 2 contact matrices, assuming that 1 million persons could be vaccinated in either 3 or 6 months. We assessed these vaccine optimization strategies for the initial strain, followed by a sensitivity analysis for the Delta variant. We found that vaccinating persons <25 years of age was associated with the lowest cumulative infections for the main matrix, for both the initial strain and the Delta variant. Prioritizing the elderly (≥65 years of age) was associated with the lowest cumulative deaths for both strains in all scenarios. The consensus between the findings of both contact matrices depended on the vaccine rollout period and the objective of the vaccination program.

Ghana reported its first case of COVID-19 on March 12, 2020, and 171,065 cases and 1,445 deaths had been recorded as of December 31, 2022 (*1*). The country introduced various public health measures when the pandemic first emerged, including school closures, travel bans, mask mandates, and, later, vaccination, all of which were associated with a decline in transmission (*2*). Ghana was the first country to receive 600,000 doses of the Oxford-AstraZeneca COVID-19 AZD1222 vaccine (https://www.astrazeneca.com) on February 24, 2021, through the COVAX program (*3*). The vaccination program was deployed in March 2021; politicians and civil society leaders publicly received vaccines to boost nationwide trust in the program (*4*). The first batch of vaccines was delivered to regions and populations with the highest burden of COVID-19: the Greater Accra and Ashanti regions, frontline healthcare workers, the elderly, and persons with comorbidities. In addition to the initially dispersed vaccine doses, the Ministry of Health received an additional supply of the AZD1222 vaccine and the Pfizer-BioNTech BNT162b2 vaccine (https://www.pfizer.com) from several high-income countries (*5*).

Given the limited availability of doses and vaccine hesitancy, only 9.2% of the Ghana population of 30,800,000 was fully vaccinated as of December 2021 (*6*). Hence, the government’s goal to reach widespread vaccine coverage by October 2021 was not met (*3*). Studies of COVID-19 vaccine hesitancy among residents of Ghana reported that >35% of study participants said they would not receive the vaccine because of concerns about vaccine efficacy and conspiracy theories (*4*,*7*). Moreover, a seroprevalence study in August 2020 found that only 19% of Ghana residents tested positive for SARS-CoV-2 IgM, IgG, or both (*8*). Such studies suggest that most of the population in Ghana remains susceptible to SARS-CoV-2, and mitigating the pandemic might best be achieved through effectively prioritizing the dispensation of limited vaccines. To optimize Ghana’s vaccination strategy, provide evidence of the benefits of vaccination, and increase uptake in the population, research is required to quantify the vaccine’s effect on the magnitude of the epidemic peak, cumulative infections, and deaths in the context of limited vaccine supplies and logistical barriers.

Several mathematical modeling studies of COVID-19 vaccination strategies in other countries and jurisdictions were published in 2020 and 2021 (*9**–**13*). Alagoz et al. used an agent-based model to simulate the transmission dynamics of COVID-19, accounting for the proportion of the population vaccinated, vaccine capacity, and adherence to nonpharmaceutical interventions (*9*). Moghadas et al. used an agent-based model to evaluate the effect of vaccination campaigns on reducing incidence, hospitalizations, and deaths (*10*). Aside from agent-based models, homogenous-mixing and age-stratified compartmental models also have been used. Matrajt et al. used an age-stratified deterministic model, paired with optimization algorithms, for 16 age groups by varying vaccination efficacy and coverages in the population (*11*). Mumtaz et al. used an age-stratified model to assess the vaccination rollout under different vaccination coverages accounting for the decline in transmission and age-mixing matrix (*12*). Bubar et al. expanded their work further to account for contact structure, seroprevalence, and age-specific vaccine efficacy (*13*). The outcomes explored in these studies included symptomatic infections, cumulative infections and deaths, and hospitalizations, focusing mainly on high-income countries outside Africa. Thus, determining who to vaccinate first when vaccines are available and analyzing the sensitivity of modeling outputs to the choice of contact matrices are underexplored in the Africa context and in Ghana specifically.

We employed an age-stratified model to assess the effect of vaccinating 1 million persons in 3 versus 6 months using 2 Africa contact matrices. We retrospectively assessed the counterfactual effect of various age-targeted vaccine optimization strategies against the initial and Delta strains of SARS-CoV-2 when vaccines first became available. Our aim was to inform future vaccination programs by identifying factors critical to achieving optimal outcomes. The Georgia Southern University Institutional Review Board determined that this project (H20364) was exempt from full review under the nonhuman subjects determination (G8) according to the Code of Federal Regulations Title 45 Part 46.

## Methods

### Model Formulation

We proposed an age-stratified Susceptible-Exposed-Presymptomatic-Symptomatic-Asymptomatic-Recovered-Dead-Vaccinated (SEPIARD-V) model to simulate SARS-CoV-2 transmission dynamics and the effect of various vaccination scenarios (Appendix 1 Figure) (*14*). The SEPIARD-V model acknowledges that persons who are initially asymptomatic and later develop symptoms transmit the virus while in the presymptomatic phase. A 2020 study of presymptomatic transmission of SARS-CoV-2 in Singapore provided evidence of COVID-19 transmission 1–3 days before symptom onset (*15*). The model was suitable for studying the transmission dynamics of COVID-19 in Ghana because of the growing evidence that both symptomatic and asymptomatic patients transmit the infection, regardless of their symptomatic status (*16*,*17*). Our model, therefore, assumed that presymptomatic, asymptomatic, and symptomatic persons contributed to transmission. Our model also assumed that immunity from both natural infection and vaccination waned over time, making reinfection possible (Table 1; Appendix 1). An effective reproduction number (R_t_) of 3.13 was assumed for the virus in the main analysis to represent the virus strain that first hit Ghana in the spring of 2020, referred to as the initial strain in this study (Armachie et al., unpub. data, https://doi.org/10.20944/preprints202104.0125.v1). A higher R_t_ of 5.35 was assumed for the Delta variant in the scenario analyses, with a reduced vaccine efficacy of 67% for the AZD1222 vaccine (*32*; Pearson et al., unpub. data, https://doi.org/10.1101/2021.12.19.21268038). Our model was run for 500 days to allow enough time for the first wave of the epidemic to subside and to include observations relevant to when the second wave began to emerge.

**Table 1:** Parameter values for age-stratified SEPIARD-V COVID-19 model to assess health outcomes of COVID-19 vaccination strategies, Ghana*

Parameter	Symbol	Value	References
Mean latency period which is the period from exposure to infectiousness	1/k	1.85 d	Abbasi et al. (*18*), Liu et al. (*19*)
Mean duration of being infectious and symptomatic	1/f	15.7 d	Cai et al. (*20*), Xing et al. (*21*)
Mean duration of being infectious and asymptomatic	1/q	7.25 d	Ma et al. (*22*), Byrne et al. (*23*)
Mean duration of being infectious and presymptomatic	1/c	2.9 d	Tindale et al. (*24*), Byrne et al. (*23*)
Reproduction number for the initial strain	R	3.13	Armachie et al., unpub. data, https://doi.org/10.20944/preprints202104.0125.v1
Reproduction number for the Delta strain	R	5.35	Pearson et al., unpub. data, https://doi.org/10.1101/2021.12.19.21268038
Probability of exposed person becoming presymptomatically infected	δ	0.30	Chen et al. (*25*), Buitrago-Garcia et al. (*26*)
Vaccine efficacy against infection	σ	0.745	Knoll et al. (*27*)
Relative transmissibility of asymptomatic persons	u	0.75	CDC (*28*)
Relative transmissibility of presymptomatic persons	r	0.75	CDC (*28*)
Mean duration of immunity after vaccination	χ	182 d	Iacobucci (*29*)
Mean duration of immunity after natural infection	w	365 d	Good and Hawkes (*30*)
Age-specific case-fatality ratio	z	0.002 for <25 y, 0.005 for 25–64 y, 0.048 for ≥65 y	Our World in Data (January 26, 2021–November 12, 2021; *22*), Lawal (*31*)
Daily vaccination rate	v	Varied by 0.00009–0.0163977 d^–1^ per person	Estimated

*CDC, Centers for Disease Control and Prevention; SEPIARD-V, Susceptible-Exposed-Presymptomatic-Symptomatic-Asymptomatic-Recovered-Dead-Vaccinated.

### Age Groups and Contact Matrices

Because of the strong evidence of assortative mixing between age groups in sub-Saharan Africa (*33**,**34*), we incorporated a contact matrix between age groups into the model. We stratified the population was stratified into 3 groups: <25 years, 25–64 years, and ≥65 years of age. Two contact matrices were adapted from studies in Uganda (main matrix) and Ethiopia (second matrix) (*33**,**35*). The main matrix suggested that, on average, the within-group contact rate among persons <25 years of age was 23.58 per day; for persons 25–64 years of age, that contact rate was 15.05 per day; and for persons ≥65 years of age, the contact rate was 0.54 per day (*33*). For the second matrix, on average, the within-group daily contact rate was 8.2 among persons <25 years of age, 7.8 for persons 25–64 years of age, and 1.6 for persons ≥65 years of age (*35*). The population breakdown for Ghana was 56.08% <25 years of age (n = 17,272,640), 39.48% 25–64 years of age (n = 12,159,840), and 4.44% ≥65 years of age (n = 1,367,520) (*36*) (Appendix 1). 

### Scenario Analyses

Our analysis aimed to determine which age group should be prioritized in the case of limited vaccine supply under different rollout speeds. We analyzed multiple scenarios, looking at a percentage of each subpopulation vaccinated when prioritizing different age groups, with coverage calculated for 1 million people using the 2021 population (*37*). The primary scenarios, by percentage of persons vaccinated in each age group, were as follows: (i) 73.1% of persons ≥65 years of age; (ii) 8.2% of persons 25–64 years of age; (iii) 5.8% of persons <25 years of age; and (iv) 3.4% of persons <65 years of age. We also assessed projected outcomes of vaccinating each age group at the same rate without prioritization (v). We used 2 rollout speeds (daily vaccination rates) in each scenario, assuming 2 million doses can be exhausted in 3 months and 6 months (Appendix 1 Tables 1–3). Finally, we performed analyses for 2 additional scenarios by changing the assumptions on vaccine supplies. First, the number of people to be vaccinated was either halved or doubled. Hence, we assumed enough vaccines were available for 500,000 persons (1 million doses) and 2,000,000 persons (4 million doses). We repeated our age-specific scenario analysis using the second contact matrix, adapted from Trentini et al. (*35*).

### Analysis

We solved our model’s system of ordinary differential equations according to the Runge-Kutta 4 method in the deSolve package in R version 4.1.1 (The R Foundation for Statistical Computing, https://www.r-project.org). We estimated the number of infections and deaths averted in the general population and compared them across all study scenarios. We assessed the percent of the population who were symptomatic at the peak, those who were ever infected (cumulative infections), and those who died (cumulative deaths) (Appendix 1; Appendix 2).

## Results

### Symptomatic Infections at the Peak under the Main Scenario of Vaccinating 1 Million Persons

**Figure 1:**
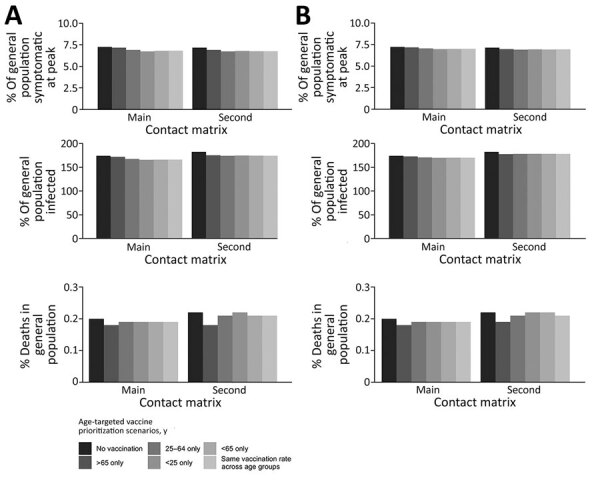
Effects of various vaccination scenarios on symptomatic infections at peak (upper panels), cumulative infections (middle panels), and deaths (lower panels) as a percentage of the general population, Ghana. The assessment used 2 different contact matrices in the main analysis and an effective reproductive number of 3.13 for the initial strain. A) Results assuming 1 million persons were vaccinated in 3 months. B) Results assuming 1 million persons were vaccinated in 6 months. Percentage of cumulative infections is >100% because of waning immunity from natural infection and vaccination.

The following results of our main analysis assumed an R_t_ of 3.13 for the initial strain. We demonstrated that vaccinating 1 million persons <25 years of age in 3 months was associated with the lowest percentage (6.75%) of symptomatic persons in the population at the peak. However, prioritizing the elderly (≥65 years of age) resulted in the highest percentage of symptomatic persons (7.19%) at the peak, given a 3-month rollout using the main matrix. If the rollout period was increased to 6 months, prioritizing persons <25 years of age also resulted in the lowest symptomatic percentages (7.01%) using the main matrix. The second matrix suggested that focusing vaccination initiatives on persons 25–64 years of age was associated with the lowest percentage of symptomatic infections (6.96%) (Tables 2, 3; Figure 1).

**Table 2:** Scenario analysis of outcomes in the total population under various vaccination scenarios, using the main matrix method for the initial strain, Ghana

Vaccine prioritization by age group, y	Scenario, % infections					
	500,000 vaccinated in 3 mo	500,000 vaccinated in 6 mo	1 million vaccinated in 3 mo	1 million vaccinated in 6 mo	2 million vaccinated in 3 mo	2 million vaccinated in 6 mo
Symptomatic infections at peak						
Only ≥65	7.22	7.24	7.19	7.22	7.16	7.19
25–64	7.09	7.17	6.92	7.09	6.61	6.92
<25	7.01	7.13	6.75	7.01	6.26	6.75
<65	7.03	7.15	6.81	7.03	6.37	6.81
Same rate across age groups	7.04	7.15	6.83	7.04	6.40	6.83
Cumulative infections						
Only ≥65	172.88	173.50	172.09	172.88	171.33	172.09
25 –64	170.80	172.57	167.44	170.80	161.00	167.44
<25	170.04	172.20	165.76	170.04	157.17	165.76
<65	170.28	172.43	166.19	170.28	158.20	166.19
Same rate across age groups	170.41	172.39	166.44	170.41	158.51	166.44
Deaths						
Only ≥65	0.18	0.19	0.17	0.18	0.17	0.17
25–64	0.19	0.19	0.19	0.19	0.18	0.19
<25	0.19	0.19	0.19	0.19	0.18	0.19
<65	0.19	0.19	0.19	0.19	0.18	0.19
Same rate across age groups	0.19	0.19	0.19	0.19	0.18	0.19

**Table 3:** Scenario analysis of outcomes in the total population under various vaccination scenarios using the second matrix method for the initial strain, Ghana

Vaccine prioritization by age group, y	Scenario, % infections					
	500,000 vaccinated in 3 mo	500,000 vaccinated in 6 mo	1 million vaccinated in 3 mo	1 million vaccinated in 6 mo	2 million vaccinated in 3 mo	2 million vaccinated in 6 mo
Symptomatic infections at peak						
Only ≥65	7.02	7.10	6.92	7.02	6.82	6.92
25–64	6.96	7.07	6.75	6.96	6.35	6.75
<25	6.99	7.09	6.79	6.99	6.42	6.79
<65	6.97	7.08	6.76	6.97	6.35	6.76
Same rate across age groups	6.98	7.08	6.76	6.98	6.35	6.76
Cumulative infections						
Only ≥65	177.71	179.61	175.36	177.71	173.19	175.36
25–64	178.22	180.28	174.26	178.22	166.42	174.26
<25	178.62	180.48	174.95	178.62	167.74	174.95
<65	178.39	180.48	174.42	178.39	166.69	174.42
Same rate across age groups	178.30	180.33	174.25	178.30	166.13	174.25
Deaths						
Only ≥65	0.19	0.20	0.18	0.19	0.17	0.18
25–64	0.21	0.22	0.21	0.21	0.19	0.21
<25	0.22	0.22	0.22	0.22	0.21	0.21
<65	0.22	0.22	0.21	0.22	0.20	0.21
Same rate across age groups	0.21	0.22	0.21	0.21	0.20	0.21

### Cumulative Infections under the Main Scenario of Vaccinating 1 Million Persons

**Figure 2:**
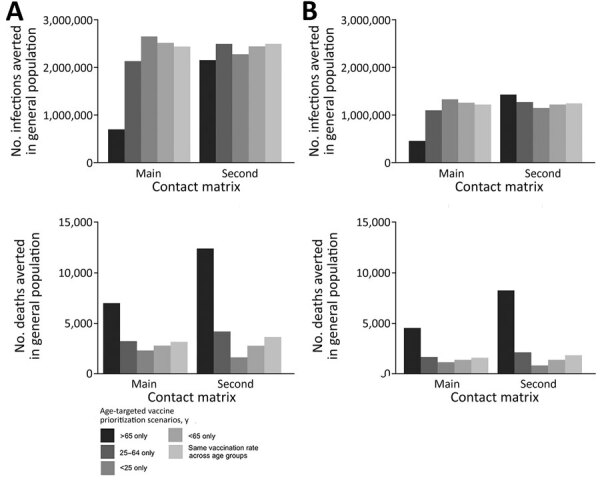
Effects of various vaccination scenarios on the number of cumulative infections averted (upper panels) and the number of deaths averted (lower panels) in the general population, Ghana. The assessment used 2 different contact matrices in the main analysis and an effective reproductive number of 3.13 for the initial strain. A) Results assuming 1 million persons were vaccinated in 3 months. B) Results assuming 1 million persons were vaccinated in 6 months.

Our results suggest that vaccinating persons <25 years of age was associated with the largest number of cumulative infections averted in Ghana under the assumption of vaccinating 1 million people in 3 months (2,653,676 cases), whereas vaccinating persons ≥65 years of age was associated with the smallest number averted (702,432 cases) (Figure 2). We also found that vaccinating persons <25 years of age should be prioritized when the population was vaccinated at a slower rate (over 6 months) or when the vaccine supply doubled or halved (Table 2). The results were sensitive to a change in the contact matrix (Table 3).

### Cumulative Deaths Averted under the Main Scenario of Vaccination of 1 Million Persons

Vaccinating the elderly (≥65 years of age) could avert >7,000 deaths if 1 million people were vaccinated over 3 months, assuming the main contact matrix, and >4,000 deaths would be averted if the elderly population was vaccinated over 6 months. The number of deaths prevented was the lowest when persons <25 years of age were prioritized in both vaccination time frames (2,317 in 3 months vs. 1,157 in 6 months), assuming the main contact matrix (Figure 2). Vaccinating the elderly (≥65 years of age) remained the best option to reduce deaths, regardless of the mixing patterns (Tables 2, 3; Figures 1, 2).

### Varying Vaccine Supply to Vaccinate 500,000 or 2 Million Persons

Similar to the base case scenarios of vaccinating 1 million persons, prioritizing persons <25 years of age was associated with the lowest percentage of cumulative infections if 500,000 or 2 million persons were vaccinated in 3 or 6 months (Table 2). However, simulations using the second matrix reported mixed results. For example, prioritizing the elderly seemed to be the best strategy for lowering cumulative infections when vaccine supplies were only enough for 500,000 people (177.71% for 3 months and 179.61% for 6 months). In contrast, vaccinating each age group at the same rate was preferred when the supply was enough to vaccinate 2 million persons (166.13% for 3 months and 174.25% for 6 months) (Table 3). Prioritizing the elderly (≥65 years of age) remained the strategy of choice to specifically lower COVID-19 mortality for both matrices (Tables 2, 3).

### Comparing Outcomes for the Initial and Delta Variants in the Absence of Vaccination

In the absence of vaccination, the scenario analysis for the Delta variant using the main matrix suggested 10.29% of symptomatic persons at the peak, 231.24% of persons having cumulative incidence of COVID-19, and 0.28% deaths in the population (Appendix 1 Table 4). Those percentages were higher than those for the initial strain, which were calculated as 7.26% of symptomatic persons at peak, 174.37% of persons having cumulative incidence of COVID-19, and 0.20% deaths in the population (Figure 1). Those findings were consistent with the results from the second matrix (Appendix 1 Table 5). In the Delta variant scenario, the percentage of symptomatic persons at the peak was slightly lower with the second matrix (10.14%) than with the main matrix (10.29%). However, cumulative infections (238.73%) and deaths (0.31%) were higher with the second matrix in the Delta variant scenario (Appendix 1 Tables 4, 5).

### Impact of Vaccination Strategies on Symptomatic Infections at the Peak Due to the Delta Variant

In analyzing vaccine prioritization for the Delta variant scenario, we found that prioritizing persons <25 years of age was associated with the lowest percentage of symptomatic infections at the peak, regardless of the available vaccine doses and rollout speed, using the main matrix (Table 4). As for the initial strain scenario, prioritizing persons <65 years of age was associated with the lowest percentage of symptomatic infections at the peak (9.89%) under the assumption of vaccinating 1 million persons over 3 months using the second matrix for the Delta variant (Table 5).

**Table 4:** Comparing outcomes for the Delta variant if 1 million persons were vaccinated under the various vaccination strategies using the main matrix method, Ghana

Vaccine prioritization by age group, y	Scenario, % infections			
	Initial strain, 3 mo	Delta variant, 3 mo	Initial strain, 6 mo	Delta variant, 6 mo
Symptomatic infections at peak				
Only ≥65	7.19	10.22	7.22	10.25
25–64	6.92	10.08	7.09	10.18
<25	6.75	9.99	7.01	10.14
<65	6.81	10.02	7.03	10.15
Same rate across age groups	6.83	10.03	7.04	10.16
Cumulative infections				
Only ≥65	172.09	228.43	172.88	229.50
25–64	167.44	227.00	170.80	229.07
<25	165.76	226.32	170.04	228.77
<65	166.19	226.50	170.28	228.87
Same rate across age groups	166.44	226.55	170.41	228.89
Deaths				
Only ≥65	0.17	0.25	0.18	0.26
25–64	0.19	0.27	0.19	0.28
<25	0.19	0.28	0.19	0.28
<65	0.19	0.27	0.19	0.28
Same rate across age groups	0.19	0.27	0.19	0.28

**Table 5:** Comparing outcomes for the Delta variant if 1 million persons were vaccinated under the various vaccination strategies using the second matrix methods, Ghana

Vaccine prioritization by age group, y	Scenario, % infections			
	Initial strain, 3 mo	Delta variant, 3 mo	Initial strain, 6 mo	Delta variant, 6 mo
Symptomatic infections at peak				
Only ≥65	6.92	9.96	7.02	10.04
25–64	6.75	9.90	6.96	10.01
<25	6.79	9.90	6.99	10.01
<65	6.76	9.89	6.97	10.01
Same rate across age groups	6.76	9.89	6.98	10.01
Cumulative infections				
Only ≥65	175.36	232.82	177.71	235.15
25–64	174.26	234.30	178.22	236.48
<25	174.95	234.44	178.62	236.57
<65	174.42	232.29	178.39	236.50
Same rate across age groups	174.25	234.13	178.30	236.43
Deaths				
Only ≥65	0.18	0.26	0.19	0.28
25–64	0.21	0.31	0.21	0.31
<25	0.22	0.31	0.22	0.31
<65	0.21	0.31	0.22	0.31
Same rate across age groups	0.21	0.31	0.21	0.31

### Impact of Vaccination Strategies on Cumulative Infections and Deaths Caused by Delta Variant

The scenario where 1 million people were vaccinated over 3 months suggested that focusing on persons <25 years of age had the lowest value of cumulative infections (226.32%) for the Delta variant, findings that paralleled our analysis of the initial strain (165.76%). Prioritizing persons <25 years of age was the best option to minimize cumulative infections in the population with a 6-month rollout for the Delta variant (228.77%) (Table 4). Importantly, the results on cumulative infections of the second matrix suggested the elderly (235.15%) should be prioritized for vaccination first for the Delta variant with a 6-month rollout (Table 5). Prioritizing the elderly remained the best strategy for lowering deaths in the population for the initial strain and the Delta variant in all the scenarios (Tables 4, 5; Appendix 1 Tables 4, 5).

## Discussion

Vaccination is the best tool to control the spread of SARS-CoV-2 and minimize the burden of COVID-19 globally. Because Ghana primarily relies on multilateral donations for their COVID-19 vaccine supply, there is a need to determine the best vaccine optimization strategies to minimize deaths, cumulative case counts, and epidemic peaks over a relatively short period. Using 2 contact matrices, we used an age-stratified mathematic model to answer the question of who should get vaccinated first when the vaccine supply is limited and when supplies are exhausted over 3 and 6 months. Our findings suggest that, for both the initial strain and the Delta variant, prioritizing persons <25 years of age for vaccination would avert the most cumulative infections and prioritizing the elderly (≥65 years of age) would result in the lowest death counts.

Optimization of vaccine prioritization strategy is sensitive to the population structure. Prioritizing younger persons to avert cumulative infections is a finding that has been reported in other studies (*13*,*38*,*39*). Bubar et al. concluded in their multicountry research that the cumulative incidence of COVID-19 was lowest when adults 20–49 years of age were prioritized, especially with a highly effective transmission-blocking vaccine (*13*). In Senegal, Diarra et al. used an age-structured dynamic mathematical model to explore various vaccination strategies and reported that prioritizing persons <60 years of age was associated with the lowest case burden (*40*). Those authors argued that countries with younger populations, such as Ghana, should prioritize vaccinating younger persons to minimize hospital costs and productivity loss.

As was the case for our team, most research teams conducting previous studies concluded that prioritizing the elderly was associated with the lowest mortality. However, Bubar et al. reported that persons 20–49 years of age should be prioritized to minimize mortality when transmission is low, when vaccine efficacy is lower in older adults, and when the vaccine is highly effective in blocking transmission. Buckner et al. reported results similar to those in our study and found that, to control deaths directly, the elderly should be vaccinated first, after stratifying young adults by essential worker status (*41*). Although the conclusions in that study and our study were similar, Buckner et al. used a dynamic approach in modeling vaccine allocation strategies that accounted for changes in the epidemiologic status of the population (shares of the population in different disease states) over 6 months using stochastic nonlinear programming techniques. In a vaccine optimization modeling study in India, Foy et al. concluded that prioritizing older adults (>60 years of age) was associated with the most significant reduction in deaths, regardless of vaccine efficacy, control measures, and rollout speed (*38*). Another modeling study by Chapman et al., using COVID-19 data from California, reported similar results (*42*); however, that study focused on identifying the groups to prioritize after healthcare workers and long-term facility residents received initial vaccine doses.

The differences in outcomes between the contact matrices in our study might be due to the much lower reported contact rates among the younger population in the matrix adapted from Trentini et al. (i.e., second matrix) (*35*). The study by Bubar et al. on vaccine optimization strategies across multiple countries, including South Africa, concluded the best vaccination strategy depended on the extent of mixing patterns (*13*). The use of 2 contact matrices reflects the degree to which decision makers should consider social interactions in the population before optimizing vaccination strategies when vaccine supplies are limited. Our findings demonstrate that the mixing pattern is relevant when the goal of the vaccination program is to minimize infection burden and the vaccine rollout takes place over an extended period. Thus, a population with lower contact rates among the older population would need to prioritize younger persons. However, contact patterns in the population may not be relevant if the goal of the vaccination program is to minimize deaths and vaccine uptake is high. Future studies might consider exploring differences observed using matrices of different settings; for example, rural versus urban and household versus community mixing.

As reported by Ko et al., the question of who should receive vaccinations first depends also on the objective for vaccination (minimizing cumulative infections or deaths) and the effective reproduction number (*39*). Thus, policymakers might need to consider compromises in deciding the best vaccine allocation strategies. For example, prioritizing the elderly may lead to fewer deaths but higher case numbers, which could exacerbate economic loss due to a high case count in a younger population. The transmissibility of the circulating variant also might inform a vaccine optimization strategy. We did not see evidence of this effect in our study because the priority group remained the same for the Delta variant, which carried a higher reproduction number. Another study concluded vaccine optimization depended on the vaccine supply (*42*).

Although our study demonstrates the need to prioritize certain age groups to minimize the burden of COVID-19 in Ghana, depending on the objective of the program, other factors need to be considered to ensure people receive vaccinations when they become eligible. Employing targeted vaccine campaigns to minimize hesitancy among the prioritized group might be a necessary part of the program. Acheampong et al. reported the level of reluctance among older adults was lower than that for younger adults in Ghana (*43*). Likewise, a survey among persons >65 years of age in the United States found that 91% of the elderly were willing to get vaccinated (*44*). This reported vaccine hesitancy across different age groups suggests a need for campaigns to create an enabling environment and engage younger populations about their role in mitigating the pandemic.

The first limitation of our study is that our model was age-stratified only. Other demographic variables (e.g., occupation and comorbidity) might change a person’s COVID-19 infection risk and clinical prognosis (*45*). Second, our model did not include a hospitalization compartment. Thus, we could not evaluate the effects of the Omicron variant, for which vaccines demonstrated effectiveness against severe infections but modest effectiveness against infection. Third, the contact matrices we used were adapted from other countries in Africa. Those countries had similar demographic distributions as Ghana, and we assumed that the frequency of contact in the population would be similar. Fourth, we did not have data to represent the rural and urban differences in contact matrices in Ghana. Fifth, our model design specified the symptomatic period to be the same for persons who recovered from COVID-19 and those who died from it. Sixth, the highly transmissible Omicron variant was not included in our study because of limited evidence of the effectiveness of vaccines against infection from that variant (*46*). Last, our model accounted for vaccine effectiveness against infection but did not account for the reduction of the case-fatality ratio if a person was vaccinated and still became infected.

In conclusion, we used an age-stratified compartmental model to assess the impact of various COVID-19 vaccine allocation strategies in Ghana. Our study reiterates the need to increase vaccination rates by ensuring increased vaccine supplies and faster rollout speed. Vaccinating persons <25 years of age was associated with the highest numbers of cumulative infections averted for the initial strain and the Delta variant. Prioritizing persons ≥65 years of age was associated with the lowest deaths in the population. Our findings indicate that vaccine prioritization strategies in Ghana, or in any country, depend on the country’s policy objectives, population structure, mixing patterns, and vaccine supply.

Appendix 1 Additional information for age-stratified model to assess health outcomes of COVID-19 vaccination strategies, Ghana.

Appendix 2 Coding in R for age-stratified model to assess health outcomes of COVID-19 vaccination strategies, Ghana.
