# Supplementary material for: Age-Stratified Model to Assess Health Outcomes of COVID-19 Vaccination Strategies, Ghana
Source: Emerg Infect Dis. 2023 Feb;29(2):360–70. doi: 10.3201/eid2902.221098 (PMC9881782; doi:10.3201/eid2902.221098)
Supplement: Appendix 1 — Additional information for age-stratified model to assess health outcomes of COVID-19 vaccination strategies, Ghana. [file 22-1098-Techapp-s1.pdf]

Article DOI: http://doi.org/10.3201/eid2902.221098

Age-Stratified Model to Assess Health Outcomes of COVID-19 Vaccination Strategies, Ghana

Appendix 1

Methods

Model equations

The total population of Ghana, N, is assumed to be a constant and equals the sum of the compartments representing individuals with different disease statuses (including COVID-19-specific deaths) at any time during the simulation and that births and deaths from natural (non-COVID-19) causes do not affect the infection dynamics in the population:

$N = S + E + P + I + A + R + V + D$ (Eq. 1)

Following the assumption of a frequency-dependent model, we define the force of infection, λ, as a combination of infection terms with symptomatic, asymptomatic, and pre-symptomatic individuals, each contributing to the transmission process as follows:

$\lambda = \frac{\beta(I+uA+rP)}{N}$ (Eq. 2),

where the contributions of asymptomatic and presymptomatic individuals are fractions u and r of that of the symptomatic individuals.

The set of ordinary differential equations (ODE) that defines the progression of susceptible individuals through different disease statuses upon infection and a vaccinated and immune status upon vaccination and their re-entry into the susceptible state due to waning immunity is described below:

$dS/dt = (-\lambda S) + wR - (v\sigma S) + \chi V$ (Eq. 3)

$dE/dt = \lambda S - kE$ (Eq. 4)

$dP/dt = \delta kE - cP$ (Eq. 5)

$dI/dt = cP - (1-z)fI - zfI$ (Eq. 6)

$dA/dt = (1-\delta)kE - qA$ (Eq. 7)

$dR/dt = (1-z)fI + qA - wR$ (Eq. 8)

$dD/dt = zfI$ (Eq. 9)

$dV/dt = v\sigma S - \chi V$ (Eq. 10)

$dC/dt = kE$ (Eq. 11)

In the SEPIARD-V model, the population is initially susceptible until an infectious individual is introduced. After contact with an infectious person, susceptible individuals are infected at a rate of λ (force of infection). While in the latent period (E), they do not transmit the virus. Individuals leave the latent period at a rate of k and can either become asymptomatically (A) or pre-symptomatically infectious (P). Asymptomatic individuals will recover (move to the R compartment) at a rate of q without showing any symptoms (*1*,*2*). Pre-symptomatic infectious individuals become symptomatic (I) at a rate of c. The mean duration of the symptomatic period is defined as $1/f$. A fraction z of symptomatic individuals will die from COVID-19 (move to the D compartment) while the other fraction (1-z) will recover (move to the R compartment). Susceptible individuals become fully vaccinated (move to the V compartment) at a rate of v per day, while the vaccine is assumed to have an efficacy (or effectiveness) of σ.

Model parameters

In our model, after susceptible individuals are exposed, the latent period, which is the period from exposure to infectiousness, is $1/k$ and is assumed to have a mean of 1.85 days (*3*,*4*). Once exposed, a third (δ) of individuals become pre-symptomatically infected, and the rest (1-δ) become asymptomatic (*5*,*6*). The mean pre-symptomatic period, $1/c$, is assumed to be 2.9 days (*7*). The mean duration of infectiousness for symptomatic individuals ($1/f$) is 15.7 days, and that of asymptomatic individuals ($1/q$) is 7.25 days (*8–11*). The transmission rate, β, is estimated from the reproduction number (R) using the formula $(1-\delta)\left(\frac{u\beta}{q}\right) + \delta(\frac{r\beta}{c} + \frac{\beta}{f})$ (*12*), assuming a value of 3.13 for the initial strain as assessed by Armachie and colleagues (unpub. data, https://doi.org/10.20944/preprints202104.0125.v1). This value would be updated in our scenario analysis of the delta variant. As we assumed some individuals were recovered and temporarily immune at the beginning of the simulation (see 'Model initialization' below), the transmission

rate was derived from an effective reproductive number of 3.13 for the initial strain and 5.35 for the Delta variant respectively. According to the CDC COVID-19 pandemic planning scenarios, the relative transmissibility of asymptomatic and pre-symptomatic individuals, u and r, are assumed to be 0.75, respectively (*13*). Two doses of the AstraZeneca COVID-19 vaccine were reported to have an efficacy (σ) of 0.745 (*14*). This value would also be updated in our scenario analysis of the delta variant. Immunity is acquired from either natural infection or vaccination. Vaccination-induced immunity offers protection from infection for six months (180 days) and wanes at a rate of χ, while that from natural infection, w, is about one year (365 days) (*15*). The rate of vaccination, v, is varied depending on the scenario. Once immunity wanes, individuals move back to the susceptible compartment. Details of model parameters are found in Appendix 1 Table 1.

Age-stratification

The aforementioned SEPIARD-V model was further developed into an age-stratified model.

The idea of the age-stratified model was adapted from a modeling study by Keeling and White on vaccination strategies with an optimal number of cases and severity effects during Britain's 2009 H1N1 influenza pandemic (*16*). Our analysis would answer research questions similar to the Keeling and White study and include modifications to address issues pertinent to the COVID-19 pandemic in Ghana. With vaccine supplies available, policymakers would be interested in which epidemiological goal the vaccine would most impact.

A recent retrospective cohort study in Ghana by Ashninyo et al. reported that COVID-19 disproportionately affected the younger population with a mean age of 37.9 years, with the majority (56.64%) between 31 and 64 years (*17*). According to Ghana's demographics, 56.08% of the population is below 25 years, and 4.44% are 65 years or above (*18*). Therefore, the population was stratified into three groups: <25 years, 25-64 years, and 65+ years.

Age-stratified model formulation

An age-stratified compartmental model assumes that population mixing is not homogeneous and the numbers of contact between members of age groups follow a specified contact matrix. The number of secondary cases caused by an infectious individual in a totally susceptible population is commonly known as the basic reproduction number. In the context of a heterogeneous-mixing model, the basic reproduction number is also known as a basic reproductive ratio and is the

largest eigenvalue of the next generation matrix (NGM) (*19*). Following the work of Towers and Feng (*20*), the reproduction number of an age-stratified model is equal to the product of the transmission coefficient β, the mean duration of infectiousness, and the largest eigenvalue of a matrix M that is defined by its elements $M_{ij} = C_{ij}\left(\frac{N_i}{N_j}\right)$, where C_{ij} is the contact matrix, and N_i and N_j are the numbers of individuals in age groups i and j respectively (*21*).

Contact matrices used

Due to the strong evidence of assortative mixing between age groups in the general population of Uganda (*22*) and Kenya (*23*), the contact matrix of the population was considered in the modeling of vaccination allocation strategies in Ghana. As reported by Waroux and colleagues, the contact patterns of Uganda were adopted in this study because their matrix corresponds to the population groups used in this study (below 25 years, 25-64 years, and 65 years or above). There is also a similarity in the proportion of age structure between Uganda and Ghana. Waroux and colleagues used the survey method to study the contact patterns of residents in rural Uganda in 2014 and found that, on average, the within-group contact rate among individuals below 25 years is 23.58 per day; for those between 25-64 years, it was 15.05 per day and 0.54 per day for those above 64 years (*22*).

Therefore, the 3 by 3 contact matrix is:

	**Below 25 y**	**25–64 y**	**65 y and above**
**Below 25 y**	23.58	9.31	0.87
**25–64 y**	13.01	15.05	1.53
**65 y and above**	2.29	2.44	0.54

This contact matrix was corrected for reciprocity using methods described by Melegaro et al. in their study in Zimbabwe (*24*).

The second matrix was adapted from a study in Ethiopia by Trentini et al., who also used survey-type interviews to estimate age-specific patterns (*25*). The contact matrix was used due to the similar population structure to Ghana. Furthermore, the data on contact patterns were collected in 2019, prior to the COVID-19 pandemic, and may reflect recent contact rates. On average, the within-group contact rate among individuals below 25 years is 8.2 per day; for those between 25-64 years, it was 7.8 per day, and 1.6 per day for those above 64 years (*22*). The 3 by 3 contact matrix is:

	Below 25 y	25–64 y	65 y and above
**Below 25 y**	8.2	5	1
**25–64 y**	2	7.8	2.8
**65 y and above**	0.1	2.2	1.6

This contact matrix was then corrected for reciprocity using methods described by Melegaro in their study in Zimbabwe (*26*).

Case-fatality ratio in the age-stratified model

The age-specific fatality ratios were calculated using data from Odikro and colleagues' study on the epidemiology of COVID-19 outbreak in Ghana (*27*). Using the total number of cases reported in their study (n=17,763) and the percentage of cases reported in each 10-year age group as of June 30, 2020, we calculated the percentage of cases in each age group as 23.85% for persons below 25 years, 70.65% for those between 25-64 years and 5.5% for 65+ years. For the cases reported among persons 20-29 years, we assumed that half of them occurred in persons between 20-24 years, and the other half occurred in those between 25-29 years. Next we calculated the expected number of cases for <25 years (n=4,236), 25-64 years (n=12,550) and elderly (n=977). The expected number of deaths was estimated for each age group assuming that 9% of the total deaths (n=117) deaths occurred among <25 years, 51% for 25-64 years and, 40% among the elderly (65+) (*27,28*). Finally, we calculated the age-specific case fatality ratios as the ratio between the number of deaths in each age group by the number of cases in each age group. Hence, the estimates were 0.002 for <25 years, 0.005 for 25-64 years and 0.048 for 65+ years. All other variables except the vaccination rate remained the same as described in Appendix 1 Table 1.

Model initialization

The model's system of ODE was solved following the Runge-Kutta 4 method in the deSolve package in R version 4.1.1 (R Core Team; https://www.r-project.org/). To keep it simple, the population size of Ghana, N, was set to 30,800,000. We also assumed that for the base case scenario, at the beginning of the simulation, I =1, A=0, P=0, D=0, and V=0. We accounted for the age-specific seroprevalence of SARS-CoV-2 using estimates from Quarshie and colleagues in August 2020 (*29*). We, therefore, assumed that 17.5% of persons below 25 years, 43.6% of those between 25-64 years, and 18% of 65+ persons had been infected at the beginning of the simulation. These individuals were in the recovery compartment at the beginning of the

simulation. The model was run for 500 days to allow enough time for the first wave of the epidemic to die out and observe when the second wave began to emerge.

Outcomes

The cumulative number of infections and deaths averted in the general population was estimated and compared for each scenario. Furthermore, the percent of the population who were symptomatic at the peak, ever infected (cumulative infections), and cumulative deaths were assessed. The percentage of cumulative infection could exceed 100% because as immunity waned, individuals would become susceptible again to repeated infections.

R code

The R code used for simulation in this study is provided in Appendix 2.

Appendix 1 Table 1: Daily vaccination rates for vaccinating 1 million people in 3 months and 6 months using an age-stratified model

Scenario	Fraction of total population	Number in each subgroup	1 million people can be vaccinated, % of the subpopulation vaccinated	The daily vaccination rate for a campaign of 3 m	The daily vaccination rate for a campaign of 6 m
Only ≥65 y	0.0444	1,367,520	73.1%	0.00812	0.00406
25–64 y	0.3948	12,159,840	8.2%	0.00091	0.00046
<25 y	0.5608	17,272,640	5.8%	0.00064	0.00032
<65 y	0.9556	29,432,480	3.4%	0.00038	0.00019
Same vaccination rate	1	30,800,000	3.2%	0.00036	0.00018

Appendix 1 Table 2: Daily vaccination rates for vaccinating 500,000 people in 3 months and 6 months using an age-stratified model

Scenario	Fraction of total population	Number in each subgroup	500,000 people can be vaccinated, % of the subpopulation vaccinated	The daily vaccination rate for a campaign of 3 m	The daily vaccination rate for a campaign of 6 m
Only ≥65 y	0.0444	1,367,520	36.6%	0.00406	0.00203
25–64 y	0.3948	12,159,840	4.1%	0.00046	0.00023
<25 y	0.5608	17,272,640	2.9%	0.00032	0.00016
<65 y	0.9556	29,432,480	1.7%	0.00019	0.00009
Same vaccination rate	1	30,800,000	1.6%	0.00018	0.00009

Appendix 1 Table 3: Daily vaccination rates for vaccinating two million people in three months and six months using an age-stratified model

Scenario	Fraction of total population	Number in each subgroup	2 million people can be vaccinated, % of the subpopulation vaccinated	The daily vaccination rate for a campaign of 3 m	The daily vaccination rate for a campaign of 6 m
Only ≥65 y	0.0444	1,367,520	146.3%	0.01625	0.00812
25–64 y	0.3948	12,159,840	16.4%	0.00183	0.00091
<25 y	0.5608	17,272,640	11.6%	0.00129	0.00064
<65 y	0.9556	29,432,480	6.8%	0.00075	0.00038
Same vaccination rate	1	30,800,000	6.5%	0.00072	0.00036

Appendix 1 Table 4: Sensitivity analysis of outcomes in the total population under various vaccination scenarios using the main matrix for the delta variant*

Vaccine prioritization, y	500,000 people were vaccinated in 3 m, %	500,000 people were vaccinated in 6 m, %	1 million people were vaccinated in 3 m, %	1 million people were vaccinated in 6 m, %	2 million people were vaccinated in 3 m, %	2 million people were vaccinated in 6 m, %
	**Symptomatic infections at peak**					
Only ≥65	10.25	10.27	10.22	10.25	10.18	10.22
25–64	10.18	10.24	10.08	10.18	9.89	10.08
<25	10.14	10.21	9.99	10.14	9.69	9.99
<65	10.15	10.23	10.02	10.15	9.77	10.02
Same vaccination rate	10.16	10.22	10.03	10.16	9.78	10.03
	**Cumulative infections**					
Only ≥65	229.50	230.26	228.43	229.50	227.20	228.43
25–64	229.07	230.15	227.00	229.07	222.89	227.00
<25	228.77	230.00	226.32	228.77	221.41	226.32
<65	228.87	230.11	226.50	228.87	221.90	226.50
Same vaccination rate	228.89	230.07	226.55	228.89	221.89	226.55
	**Deaths**					
Only ≥65	0.26	0.27	0.25	0.26	0.23	0.25
25–64	0.28	0.28	0.27	0.28	0.27	0.27
<25	0.28	0.28	0.28	0.28	0.27	0.28
<65	0.28	0.28	0.27	0.28	0.27	0.27
Same vaccination rate	0.28	0.28	0.27	0.28	0.27	0.27

*If there were no vaccination, 10.29% of the population would be symptomatic at the epidemic peak, there would be a total of 231.24% cumulative incidence, and 0.28% of the population would die of COVID-19.

Appendix 1 Table 5: Sensitivity analysis of outcomes in the total population under various vaccination scenarios using the second matrix for the Delta variant*

Vaccine prioritization, y	500,000 people were vaccinated in 3 m, %	500,000 people were vaccinated in 6 m, %	1 million people were vaccinated in 3 m, %	1 million people were vaccinated in 6 m, %	2 million people were vaccinated in 3 m, %	2 million people were vaccinated in 6 m, %
	Symptomatic infections at peak					
Only ≥65	10.04	10.09	9.96	10.04	9.85	9.96
25–64	10.01	10.08	9.89	10.01	9.66	9.89
<25	10.01	10.08	9.89	10.01	9.66	9.89
<65	10.01	10.08	9.89	10.01	9.64	9.89
Same vaccination rate	10.01	10.08	9.89	10.01	9.64	9.89
	Cumulative infections					
Only ≥65	235.15	236.74	232.82	235.15	230.15	232.81
25–64	236.48	237.60	234.30	236.48	229.96	234.30
<25	236.57	237.65	234.44	236.57	230.18	234.44
<65	236.51	237.67	234.29	236.51	229.99	234.29
Same vaccination rate	236.43	237.58	234.13	236.43	229.52	234.13
	Deaths					
Only ≥65	0.28	0.30	0.26	0.28	0.24	0.26
25–64	0.31	0.31	0.31	0.31	0.30	0.31
<25	0.31	0.31	0.31	0.31	0.31	0.31
<65	0.31	0.31	0.31	0.31	0.30	0.31
Same vaccination rate	0.31	0.31	0.31	0.31	0.30	0.31

*If there were no vaccination, 10.14% of the population would be symptomatic at the epidemic peak, there would be a total of 238.73% cumulative incidence, and 0.31% of the population would die of COVID-19.

References

1. Liu R, Leung RK, Chen T, Zhang X, Chen F, Chen S, et al. The Effectiveness of Age-Specific Isolation Policies on Epidemics of Influenza A (H1N1) in a Large City in Central South China. PLoS One. 2015;10:e0132588. PubMed https://doi.org/10.1371/journal.pone.0132588

2. Zhao Z-Y, Zhu Y-Z, Xu J-W, Hu S-X, Hu Q-Q, Lei Z, et al. A five-compartment model of age-specific transmissibility of SARS-CoV-2. Infectious Diseases of Poverty. 2020;9(1):117.

3. Abbasi Z, Zamani I, Mehra AHA, Shafieirad M, Ibeas A. Optimal Control Design of Impulsive SQEIAR Epidemic Models with Application to COVID-19. Chaos, Solitons & Fractals. 2020;139:110054.

4. Liu Z, Magal P, Seydi O, Webb G. A COVID-19 epidemic model with latency period. Infect Dis Model. 2020;5:323–37. PubMed https://doi.org/10.1016/j.idm.2020.03.003

5. Chen M, Li M, Hao Y, Liu Z, Hu L, Wang L. The introduction of population migration to SEIAR for COVID-19 epidemic modeling with an efficient intervention strategy. Inf Fusion. 2020;64:252–8. PubMed https://doi.org/10.1016/j.inffus.2020.08.002

6. Buitrago-Garcia D, Egli-Gany D, Counotte MJ, Hossmann S, Imeri H, Ipekci AM, et al. Occurrence and transmission potential of asymptomatic and presymptomatic SARS-CoV-2 infections: A living systematic review and meta-analysis. PLoS Med. 2020;17:e1003346. PubMed https://doi.org/10.1371/journal.pmed.1003346

7. Tindale LC, Stockdale JE, Coombe M, Garlock E, Lau WYV, Saraswat M, et al. Evidence for transmission of COVID-19 prior to symptom onset. eLife. 9:e57149 https://doi.org/10.7554/eLife.57149

8. Cai Q, Huang D, Ou P, Yu H, Zhu Z, Xia Z, et al. COVID-19 in a designated infectious diseases hospital outside Hubei Province, China. Allergy. 2020;75:1742–52. PubMed https://doi.org/10.1111/all.14309

9. Byrne AW, McEvoy D, Collins AB, Hunt K, Casey M, Barber A, et al. Inferred duration of infectious period of SARS-CoV-2: rapid scoping review and analysis of available evidence for asymptomatic and symptomatic COVID-19 cases. BMJ Open. 2020;10:e039856. PubMed https://doi.org/10.1136/bmjopen-2020-039856

10. Thiruvengadam G, Ramanujam R, Marappa L. Modeling the recovery time of patients with coronavirus disease 2019 using an accelerated failure time model. J Int Med Res. 2021;49:3000605211040263. PubMed https://doi.org/10.1177/03000605211040263

11. Xing Y-H, Ni W, Wu Q, Li W-J, Li G-J, Wang W-D, et al. Prolonged viral shedding in feces of pediatric patients with coronavirus disease 2019. J Microbiol Immunol Infect. 2020;53:473–80. PubMed https://doi.org/10.1016/j.jmii.2020.03.021

12. University of British Columbia Department of Zoology. COVID-19 Models Lecture. 2020 [cited November 15, 2020]; Available from: https://www.zoology.ubc.ca/~bio301/Bio301/Lectures/Lecture1/COVID_Models.pdf

13. Centers for Disease Control and Prevention. Pandemic Planning Scenarios. 2020 [cited November 12, 2020]; Available from: https://www.cdc.gov/coronavirus/2019-ncov/hcp/planning-scenarios.html

14. Knoll MD, Wonodi C. Oxford-AstraZeneca COVID-19 vaccine efficacy. Lancet. 2021;397:72–4. PubMed https://doi.org/10.1016/S0140-6736(20)32623-4

15. Good MF, Hawkes MT. The Interaction of Natural and Vaccine-Induced Immunity with Social Distancing Predicts the Evolution of the COVID-19 Pandemic. MBio. 2020;11:e02617-20. PubMed https://doi.org/10.1128/mBio.02617-20

16. Keeling MJ, White PJ. Targeting vaccination against novel infections: risk, age and spatial structure for pandemic influenza in Great Britain. J R Soc Interface. 2011;8:661–70. PubMed https://doi.org/10.1098/rsif.2010.0474

17. Ashinyo ME, Duti V, Dubik SD, Amegah KE, Kutsoati S, Oduro-Mensah E, et al.. Clinical characteristics, treatment regimen and duration of hospitalization among COVID-19 patients in Ghana: a retrospective cohort study. Pan Afr Med J. 2020;37(Suppl 1):9. PubMed https://doi.org/10.11604/pamj.supp.2020.37.1.25718

18. Central Intelligence Agency. The world factbook. 2022 [cited December 4, 2022]. https://www.cia.gov/the-world-factbook/

19. Anderson RM, May RM. Infectious diseases of humans: dynamics and control: Oxford university press; 1992.

20. Towers S, Feng Z. Social contact patterns and control strategies for influenza in the elderly. Math Biosci. 2012;240:241–9. PubMed https://doi.org/10.1016/j.mbs.2012.07.007

21. Towers S. SIR infectious disease model with age classes. 2012 [cited September 15, 2021]. http://sherrytowers.com/2012/12/11/sir-model-with-age-classes/

22. le Polain de Waroux O, Cohuet S, Ndazima D, Kucharski AJ, Juan-Giner A, Flasche S, et al. Characteristics of human encounters and social mixing patterns relevant to infectious diseases spread by close contact: a survey in Southwest Uganda. BMC Infect Dis. 2018;18(1):172.

23. Kiti MC, Kinyanjui TM, Koech DC, Munywoki PK, Medley GF, Nokes DJ. Quantifying age-related rates of social contact using diaries in a rural coastal population of Kenya. PLoS One. 2014;9:e104786. PubMed https://doi.org/10.1371/journal.pone.0104786

24. Melegaro A, Jit M, Gay N, Zagheni E, Edmunds WJ. What types of contacts are important for the spread of infections?: using contact survey data to explore European mixing patterns. Epidemics. 2011;3:143–51. PubMed https://doi.org/10.1016/j.epidem.2011.04.001

25. Trentini F, Guzzetta G, Galli M, Zardini A, Manenti F, Putoto G, et al. Modeling the interplay between demography, social contact patterns, and SARS-CoV-2 transmission in the South West Shewa Zone of Oromia Region, Ethiopia. BMC Med. 2021;19:89. PubMed https://doi.org/10.1186/s12916-021-01967-w

26. Melegaro A, Del Fava E, Poletti P, Merler S, Nyamukapa C, Williams J, et al. Social contact structures and time use patterns in the Manicaland Province of Zimbabwe. PLoS One. 2017;12:e0170459. PubMed https://doi.org/10.1371/journal.pone.0170459

27. Kenu E, Odikro MA, Malm KL, Asiedu-Bekoe F, Noora CL, Frimpong JA, et al. Epidemiology of COVID-19 outbreak in Ghana, 2020. Ghana Med J. 2020;54(Suppl):5–15. PubMed https://doi.org/10.4314/gmj.v54i4s.3

28. Lawal Y. Africa's low COVID-19 mortality rate: A paradox? Int J Infect Dis. 2021;102:118–22. PubMed https://doi.org/10.1016/j.ijid.2020.10.038

29. Quashie PK, Mutungi JK, Dzabeng F, Oduro-Mensah D, Opurum PC, Tapela K, et al. Trends of severe acute respiratory syndrome coronavirus 2 (SARS-CoV-2) antibody prevalence in selected regions across Ghana. Wellcome Open Res. 2021; 6:173. https://doi.org/10.12688/wellcomeopenres.16890.1

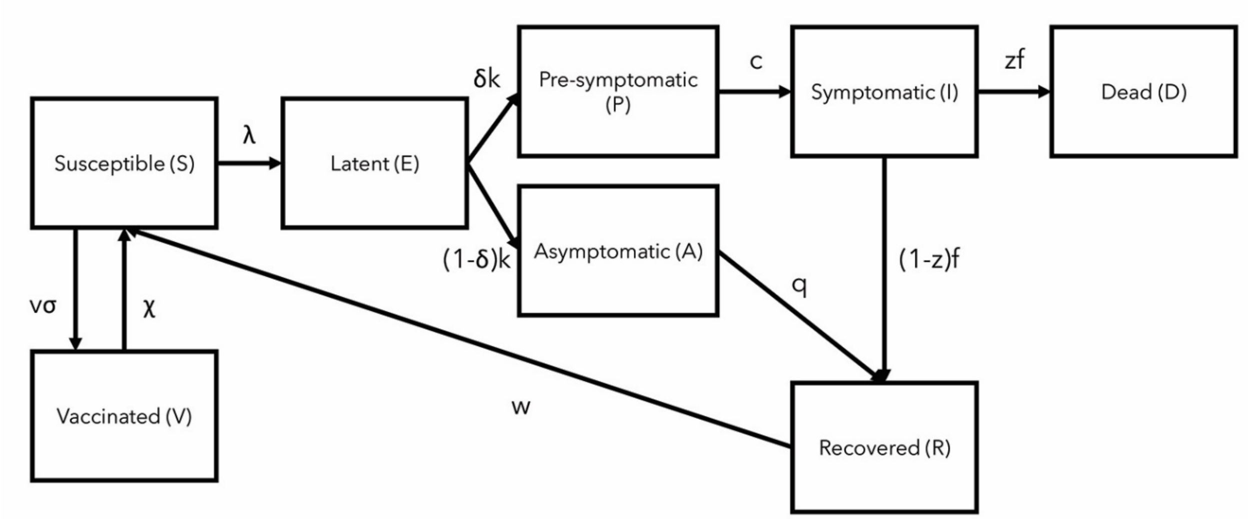

Appendix 1 Figure: The Susceptible-Exposed-Presymptomatic-Symptomatic-Asymptomatic-Recovered-Dead-Vaccinated (SEPIARD-V) model represents SARS-CoV-2 transmission and COVID-19 disease progress and the vaccination against COVID-19. Note: Age-stratification is not represented in this flow diagram.
