# Supplementary material for: Age-Stratified Model to Assess Health Outcomes of COVID-19 Vaccination Strategies, Ghana
Source: Emerg Infect Dis. 2023 Feb;29(2):360–70. doi: 10.3201/eid2902.221098 (PMC9881782; doi:10.3201/eid2902.221098)
Supplement: Appendix 2 — Coding in R for age-stratified model to assess health outcomes of COVID-19 vaccination strategies, Ghana. [file 22-1098-Techapp-s2.pdf]

Article DOI: http://doi.org/10.3201/eid2902.221098

Age-Stratified Model to Assess Health Outcomes of COVID-19 Vaccination Strategies, Ghana

Appendix 2

Coding in R for age-stratified model to assess health outcomes of COVID-19 vaccination strategies, Ghana.

First we clear the workspace to get rid of leftover variables.

```
rm(list=ls())
```

Next we clear all graphic windows.

```
graphics.off();
```

We load packages that are required for the R simulation.

```
pacman::p_load(ggplot2,
               deSolve,
               reshape2)
```

We set a seed so that the same simulation outputs can be obtained when simulations are repeated.

```
set.seed(1234)
```

SEAPIRD-V model

- S refers to susceptible individuals.
- E refers to exposed individuals who are in the latent state.
- A refers to asymptomatic individuals: A percentage of the infected population makes transitions into the asymptomatic state. They are infectious.
- P refers to pre-symptomatic individuals: A percentage of the infected population makes transitions into the pre-symptomatic state. They are infectious.
- I refers to symptomatic individuals. They are infectious.
- R refers to recovered: Asymptomatic and Symptomatic individuals enter the recovery state.
- D refers to symptomatic individuals who died from COVID-19.

- V refers to individuals who become immune from COVID-19 infection after receiving two doses of the AstraZeneca vaccine.

Parameter definitions

Parameters:

- c: transition rate from presymptomatic to symptomatic= 1/(incubation period - latency period)
- beta: transmission coefficient
- q: recovery rate for asymptomatic individuals
- delta: probability that exposed persons become presymptomatically infected
- f: the inverse of the duration of individuals being symptomatic (equals to the recovery rate for symptomatic individuals)
- u: relative transmissibility of asymptomatic individuals
- r: relative transmissibility of pre-symptomatic individuals
- sigma: vaccine efficacy
- v: vaccination rate
- chi: waning immunity of vaccinated individuals
- Ro: reproduction number
- z: age-specific case fatality ratio

In this study, two strains of COVID-19 were assessed by varying the reproduction numbers and vaccine efficacy with the values below

```
#values for R and vaccine efficacy for each strain
#initial
#Ro    = 3.13
#sigma = 0.745

#delta variant
#Ro    = 5.35
#sigma = 0.67
```

An age-specific case fatality ratio was calculated: We assumed 9% of deaths in the population occurred in children, 51% between 25 and 64 years, and 40% of deaths in the elderly. These translated into the following CFR: <25 yr = 0.002; 25-64 yr = 0.005; 65+ yr = 0.048. See Appendix 1 for more details.

```
z= c(0.002, 0.005, 0.048)
```

We created a function for the age-stratified model with 9 compartments with the set of ordinary differential equations that defines the model.

```
agestratified <- function(time,state,parameters){
  ncompartment = 9  #number of compartments in the model
  nage = length (state)/ncompartment
  S    = as.matrix(state[1:nage])
```

```
  E     = as.matrix(state[(nage+1):(2*nage)])
  A     = as.matrix(state[(2*nage+1):(3*nage)])
  P     = as.matrix(state[(3*nage+1):(4*nage)])
  I     = as.matrix(state[(4*nage+1):(5*nage)])
  R     = as.matrix(state[(5*nage+1):(6*nage)])
  D     = as.matrix(state[(6*nage+1):(7*nage)])
  V     = as.matrix(state[(7*nage+1):(8*nage)])
  L     = as.matrix(state[(8*nage+1):(9*nage)])

  I[I<0] = 0

  with(as.list(parameters),{
    N      = S+E+P+I+A+R+D+V
    lambda = (I+u*A+r*P)/N
    dS  = -beta*as.matrix(S)*(as.matrix(C)%*%(as.matrix(lambda)))+
      w*as.matrix(R)- v*sigma*as.matrix(S) + chi*as.matrix(V)
    dE  = beta*as.matrix(S)*(as.matrix(C)%*%as.matrix(lambda))- k*as.matrix(E)
    dA  = (1-delta)*k*as.matrix(E)-q*as.matrix(A)
    dP  = delta*k*as.matrix(E)-c*as.matrix(P)
    dI  = c*as.matrix(P)-f*as.matrix(I)
    dR  = (1-z)*f*as.matrix(I)+q*as.matrix(A)-w*as.matrix(R)
    dD  = z*f*as.matrix(I)
    dV  = v*sigma*as.matrix(S)-chi*as.matrix(V)
    dL  = k*as.matrix(E)

    out=c(dS, dE, dA, dP, dI, dR, dD, dV, dL)
    list (out)
  }
  )
}
```

We determined the number of persons in each age group assuming a population size of 30,800,000.

```
lcalculate_transmission_probability = 1
popul= 30800000
g= c(0.5608, 0.3948, 0.0444) #3 age classes explained above
N= popul*g #number in each age class
nage = length (g)
```

Setting initial state for compartments

We assumed a % of each subpopulation was already infected, hence they were in the recovered compartment at the beginning of the simulation.

```
I_0 = rep (1, nage) #One infectious person in each age group
S_0 = N-R_0 #susceptible individuals at the beginning of simulation in each c
lass
E_0 = rep (0, nage)
A_0 = rep (0, nage)
```

```
P_0 = rep (0, nage)
R_0 = N*(c(0.175,
          0.436,
          0.180))#accounting for initially infected in each class
D_0 = rep (0, nage)
V_0 = rep (0, nage)
L_0 = rep (0, nage)
```

Two contact matrices were used in the study a) the first matrix is defined as the "main matrix".

```
C = matrix(0, nrow=nage, ncol=nage)
C[1,1] = 23.58
C[1,2] =  9.31
C[1,3] =  0.87
C[2,1] = 13.01
C[2,2] = 15.05
C[2,3] =  1.53
C[3,1] =  2.29
C[3,2] =  2.44
C[3,3] =  0.54
```

b) The other matrix is defined as "second matrix"

```
#C = matrix(0, nrow=nage, ncol=nage)
#C[1,1] = 8.2
#C[1,2] = 5
#C[1,3] = 1
#C[2,1] = 2
#C[2,2] = 7.8
#C[2,3] = 2.8
#C[3,1] = 0.1
#C[3,2] = 2.2
#C[3,3] = 1.60
```

We set the parameter values used for calculating beta.

```
delta = 0.3
k     = 0.54
c     = 0.344
q     = 0.14
f     = 0.064
u     = 0.75
r     = 0.75
```

We defined the formulae for calculating the beta using the reproduction number from the largest eigenvalue.

```
if (lcalculate_transmission_probability==1){
  M      = C
  M[1,1] = C[1,1]*g[1]/g[1]
  M[1,2] = C[1,2]*g[1]/g[2]
```

```
  M[1,3] = C[1,3]*g[1]/g[3]
  M[2,1] = C[2,1]*g[2]/g[1]
  M[2,2] = C[2,2]*g[2]/g[2]
  M[2,3] = C[2,3]*g[2]/g[3]
  M[3,1] = C[3,1]*g[3]/g[1]
  M[3,2] = C[3,2]*g[3]/g[2]
  M[3,3] = C[3,3]*g[3]/g[3]
  eig = eigen(M)
  # Re() function extracts the real part of a complex number, in the event th
at the eigenvalue is a complex number.
  Beta = ((Ro*c*f*q)/((delta*r*f*q)+(delta*c*q)+((1-delta)*u*c*f)))/max(Re(ei
g$values))
  beta=Beta
}else{
  beta = 0.34
}
```

We defined the initial state conditions.

```
initial_state = c(S= S_0,
                  E= E_0,
                  A= A_0,
                  P= P_0,
                  I= I_0,
                  R= R_0,
                  D= D_0,
                  V= V_0,
                  L= L_0)
```

We created a vector for the parameters for initializing our model.

```
parameters = c( delta = 0.3,
                k     = 0.54,
                c     = 0.344,
                beta  = Beta,
                q     = 0.14,
                v     = 0.0003519,
                f     = 0.064,
                u     = 0.75,
                r     = 0.75,
                w     = 0.0055,
                chi   = 0.0027,
                sigma = 0.745,
                z     = z)
```

The model was simulated for 500 days to allow enough time for the second wave to emerge.

```
time = seq (0, 500, 1)
```

We solved the differential equations using the lsoda() function in the deSolve package:

```
teta<-as.data.frame(lsoda(initial_state,
                          time,
                          agestratified,
                          parameters))
```

We converted the data into a long format using the melt() function in the reshape2 package:

```
out_long=melt(teta,
              id="time")
tail (teta)
```

Scenario analysis of the impact of vaccine optimization strategies

Part 1: use v rates accounting for vaccine supplies only one million people can get vaccinated in 3 months

```
#v= c(0,        0,         0.00812)   #only elderly vaccinated
#v= c(0,        0.00091,   0)         #adults vaccinated
#v= c(0.00064,   0,         0)        #younger population vaccinated
#v= c(0.00038,   0.00038,   0)         #adults and younger vaccinated
#v= c(0,        0,         0)        #no vaccination
#v= c(0.00036, 0.00036, 0.00036) #same vaccination rate
```

Part 2: only one million people can get vaccinated in 6 months

```
#v= c(0,       0,       0.00406)  #only elderly vaccinated
#v= c(0,       0.00046, 0)       #adults vaccinated
#v= c(0.00032, 0,       0)       #younger population vaccinated
#v= c(0.00019, 0.00019, 0)       #adults and younger vaccinated
#v= c(0,       0,       0)       #no vaccination
#v= c(0.00018, 0.00018, 0.00018) #same vaccination rate
```

Part 3: only 500,000 get vaccinated in 3 months

```
#v= c(0,       0,       0.00406)  #only elderly vaccinated
#v= c(0,       0.00046, 0)       #adults vaccinated
#v= c(0.00032, 0,       0)       #younger population vaccinated
#v= c(0.00019, 0.00019, 0)       #adults and younger vaccinated
#v= c(0,       0,       0)       #no vaccination
#v= c(0.00018, 0.00018, 0.00018) #same vaccination rate
```

Part 4: only 500,000 get vaccinated in 6 months

```
#v= c(0,       0,       0.00203)  #only elderly vaccinated
#v= c(0,       0.00023, 0)       #adults vaccinated
#v= c(0.00016, 0,       0)       #younger population vaccinated
#v= c(0.00009, 0.00009, 0)       #adults and younger vaccinated
#v= c(0,       0,       0)       #no vaccination
#v= c(0.00009, 0.00009, 0.00009) #same vaccination rate
```

Part 5: only 2,000,000 get vaccinated in 3 months

```
#v= c(0,         0,          0.01625)     #only elderly vaccinated
#v= c(0,         0.00183,     0)         #adults vaccinated
#v= c(0.00129,    0,          0)         #younger population vaccinated
#v= c(0.00075,   0.00075,   0)          #adults and younger vaccinated
#v= c(0,         0,          0)          #no vaccination
#v= c(0.00072, 0.00072, 0.00072) #same vaccination rate
```

Part 6: only 2,000,000 get vaccinated in 6 months

```
#v= c(0,        0,         0.00812)   #only elderly vaccinated
#v= c(0,        0.00091,   0)         #adults vaccinated
#v= c(0.00064,  0,         0)         #younger population vaccinated
#v= c(0.00038,  0.00038,   0)         #adults and younger vaccinated
#v= c(0,        0,         0)         #no vaccination
#v= c(0.00036, 0.00036, 0.00036) #same vaccination rate
```

We defined parameter values for the simulation.

```
parameters = c( delta = 0.3,
                k     = 0.54,
                c     = 0.344,
                beta  = Beta,
                q     = 0.14,
                v     = v,
                f     = 0.064,
                u     = 0.75,
                r     = 0.75,
                w     = 0.0055,
                chi   = 0.0027,
                sigma = sigma,
                z     = z)
```

We solved the differential equations as:

```
teta<-as.data.frame(lsoda(initial_state,
                          time,
                          agestratified,
                          parameters))
out_long=melt(teta,id="time")
tail (teta)
```

Calculating the percentage of population with each outcome under the various vaccination scenarios

The percentage of the population who are symptomatic at the peak:

```
cat("The % of the population who are symptomatic at the peak is ",(max(teta$I
1+teta$I2+teta$I3)/popul)*100,"\n")
```

The % of population ever infected:

```
cat("The % of the population ever infected is ",(max(teta$L1+teta$L2+teta$L3)
/popul)*100,"\n")
```

The % of population who died:

```
cat("The % of the population who died is ",(max(teta$D1+teta$D2+teta$D3)/popu
l)*100,"\n")
```
